# Examination of different definitions of snacking frequency and associations with weight status among U.S. adults

**DOI:** 10.1371/journal.pone.0234355

**Published:** 2020-06-17

**Authors:** Alexandra E. Cowan, Kelly A. Higgins, Jennifer O. Fisher, Gina L. Tripicchio, Richard D. Mattes, Peishan Zou, Regan L. Bailey

**Affiliations:** 1 Department of Nutrition Science, College of Health and Human Sciences, Purdue University, West Lafayette, Indiana, United States of America; 2 Center for Chemical Regulation and Food Safety, Exponent, Inc., Washington, DC, United States of America; 3 Center for Obesity Research and Education, College of Public Health, Temple University, Philadelphia, Pennsylvania, United States of America; San Raffaele Roma Open University, ITALY

## Abstract

Snacks, while widely consumed in the United States (U.S.), do not have a standard definition, complicating research to understand associations, if any, with weight status. Therefore, the purpose of this study was to examine the association between snacking frequency and weight status using various snacking definitions that exist in the scientific literature among U.S. adults (NHANES 2013–2016; ≥20y n = 9,711). Four event-based snacking definitions were operationalized including participant-defined snacks, eating events outside of meals, and operationally defined snacks based on absolute thresholds of energy consumed (>50 kcal). Weight status was examined using body mass index (BMI), waist circumference, and sagittal abdominal diameter risk. Logistic regression models examined snacking frequency and associations with weight status. Outcomes varied by the definition of a snack employed, but the majority of findings were null. Mean energy from snacks was significantly higher among women with obesity compared to women with normal weight when a snack was defined as any event outside of a typical mealtime (i.e. other than breakfast, lunch, dinner, super, brunch), regardless of whether or not it contributed ≥50 kcal. Further investigation into ingestive behaviors that may influence the relationship between snacking frequency and weight status is needed.

## Introduction

Snacking (i.e., eating between meals) is a hallmark of the American dietary pattern, with more than 90% of adults in the United States (U.S.) reporting consumption of one or more snacks on a given day [1]. Although snacking has been prevalent for decades in the U.S., the frequency and portion sizes of snacks have increased over time [2]. This has prompted concern that snacking may potentially contribute to positive energy balance [1, 2]. Indeed, snacks currently comprise nearly one-quarter (22%) of total energy intake among adults [1]. Given the associations between excess energy intake and overweight and obesity, with attendant comorbidities such as type 2 diabetes, hypertension, and dyslipidemia [3, 4], understanding the role of snacking and weight status is of paramount public health importance.

The ability to elucidate the relationship between snacking and weight status is impeded by the lack of a consistent definition of the term “snack” or behavior “snacking” [5]. Various measures have assessed the energy content of the foods ingested [5, 6], timing of consumption [7–11], type of food [11, 12], juxtaposition to meal (also ambiguously defined) ingestion [13–15], or simple, consumer-defined categorizations [2, 11, 16–19]. These indices each capture one component of snacking behavior but do not adequately capture the phenomenon. Measurement of snack energy content alone fails to account for possible compensatory or exacerbating effects of eating frequency; in addition, defining by snack energy content can lead to defining trivial amounts of energy as snacks and categorizing high-energy events between typical meal times as meals. Timing of snack consumption does not account for portion size and/or energy density. Categorizing foods as snacks or meal components is fraught with error since many foods are commonly ingested under both eating events. Drawing a distinction between ingestive events labeled as “snacks” or “meals” requires that at least one be clearly defined and that has not been the case in research to date. Finally, the reliance on consumer-defined categorizations, often the only practical option in large epidemiological trials, suffers from uncontrolled inter-individual variance in opinion of what a snack is and reporting bias. Therefore, the snack definitions used in a dietary analysis address different research questions and have important implications for how the findings can be interpreted.

Given the limitations of each snack definition and the consequent low confidence in the conclusions that can be drawn from studies that use them [7, 20–23], there are conflicting viewpoints on whether to encourage or restrict snack consumption [18, 19, 24–27]. Some evidence indicates that snacking may contribute substantially to nutrient intake and, if appropriately managed, could enhance diet quality [25]. In order to address these limitations, the present study aims to examine associations between snacking and body weight status using multiple definitions of intake.

Previous studies have shown that snacking frequency is associated with greater risk of overweight and obesity (OW/OB) in both younger children [16] and among older children and adolescents [17]. However, compensatory responses to additional dietary energy either in the form of increased ingestive frequency or portion size tend to be more robust among children than adults in some [20, 21, 28], but not all clinical trials [22]. Therefore, increased snacking frequency may pose a greater risk of OW/OB among adults. However, little is known about the nature and ramifications of adult snacking. Therefore, the aim of this NHANES analysis was to examine associations of snacking and weight status among U.S. adults, comparing four definitions of a snack based on definitions commonly used in the literature. Measures of weight status include body mass index (BMI), as well as waist circumference (WC) and sagittal abdominal diameter (SAD); two emerging parameters related with cardio metabolic health [23, 29, 30].

## Methods

### Survey description

The 2013–2016 National Health and Nutrition Examination Survey (NHANES) was used for the present analysis. NHANES is a nationally representative, continuous cross-sectional survey of the noninstitutionalized, civilian residents of the U.S., conducted by the Centers for Disease Control and Prevention, National Center for Health Statistics (NCHS) [31]. NHANES employs a complex, stratified, multistage probability cluster sampling design. The NHANES protocol includes an in-person household interview that queries health information and demographics as well as a follow-up health examination in the Mobile Examination Center (MEC) for each participant. All demographic and health-related characteristics included in this report were collected during the household interview. Anthropometric measurements and dietary intake data were collected by trained study staff approximately three weeks after the household interview during the follow-up health examination in the MEC. Written informed consent was obtained for all participants or proxies and NHANES survey protocol was approved by the Research Ethics Review Board at NCHS. Additional details about NHANES dietary and anthropometric assessment are available elsewhere [32, 33].

Demographic, anthropometric, and dietary data from the NHANES 2013–2014 and 2015–2016 cycles were combined to form a sample of 20,146 participants. Participants who were < 20 years of age (n = 8,295), did not complete either the household interview or MEC examination (n = 789), were missing BMI or race/Hispanic origin information (n = 136), pregnant and/or lactating (n = 217), or did not provide reliable dietary recall information that met the minimum criteria (DR1DRSTZ not equal to 1) (n = 932) were excluded, yielding a total sample of 9,777 U.S. adults. Data on WC and SAD were available for 9,473 and 9,312 participants, respectively. Few participants were underweight (58 men and 86 women), and thus excluded due to small sample size, resulting in a final analytic sample of 9,633 adults (4,726 men and 4,907 women).

### Demographic and health-related characteristics

Demographic and health-related characteristics, including data on sex, age, race/Hispanic origin, income, and smoking status were collected from participants using the Computer-Assisted Personal Interview system during the household interview. These covariates were included because of their association with BMI and other dietary behaviors [34–37]. Age (in years) was included as a continuous variable in all analyses. Race and Hispanic origin is categorized in this analysis as recommended by the NCHS as non-Hispanic white, non-Hispanic black, non-Hispanic Asian, and Hispanic/Mexican American (combined). Income was classified using the family income-to-poverty ratio (PIR), a measure of income established by the Department of Health and Human Services representing the ratio of family income to the poverty guidelines relative to family size, a variable that is available in NHANES (INDFMPIR) [38]. Three PIR categories were used in this analysis: ≤ 130%, 131% to 350%, and > 350% to be consistent with previous NHANES analyses [39–42]. Current smoking status was determined based on whether participants were never smokers (smoked < 100 cig/lifetime), former smokers (>100 cig/lifetime but do not currently smoke), or current smokers. Current smokers were then further classified based on whether they smoked cigarettes daily (current, daily) or whether they classified themselves as a smoker, but did not smoke cigarettes daily (current, occasional) [39, 43].

### Dietary assessment

Dietary intake was self-reported in the MEC using the in-person, day one 24-hour dietary recall (24HR). The 24HR is collected by trained interviewers using the U.S. Department of Agriculture’s (USDA) automated multiple-pass method (AMPM) [44]. The USDA, Food and Nutrient Database for Dietary Studies 2015–2016 (FNDDS) [45] was used to convert foods and beverages as reported, to their respective energy and nutrient values. For each ingestive event reported on the 24HR, a time stamp, name of eating event, and all foods and/or beverages consumed during that event were collected. Types of ingestion events and their Spanish equivalents include: breakfast, lunch, dinner, supper, brunch, snack, drink, desayano, almuerzo, comida, merienda, cena, entre comida, botana, bocadillo, tentempie, bebida, and extended consumption. Events defined as “infant feeding,” “other,” or “don’t know” were excluded from this analysis. Consistent with previous studies, all foods consumed at the same clock time were considered the same ingestive event [6, 46]. Mean energy density of a snack was included as a covariate in the models to serve as a proxy for nutrient quality and was calculated as the total energy density of a snack (kcal/g) inclusive of foods and beverages. Energy density varies extensively between liquids and semi-sold/solid foods due to differences in water content [47]; thus, preliminary analyses calculating mean energy density with and without the inclusion of beverages were conducted. Insignificant differences in mean total snack energy density were observed when excluding beverages; therefore, mean total snack energy density inclusive of beverages was chosen as the continuous covariate in all subsequent analyses including mean energy density of a snack (Models 1 and 3, described below). Models adjusting for mean energy of a meal were also explored; however, due to strong correlation between mean energy of a meal and the ratio of reported energy intake (EI) to estimated energy requirement (EER), as observed by Murakami et al. [6], this covariate was excluded from all subsequent analyses. Additional dietary variables included in the models as nominal covariates include recall day of the week (weekday/ weekend), and typical food consumption day amount (as defined in NHANES (DR1_300)).

### Reporting accuracy

Self-reported dietary intake data can be prone to systematic error, like energy underreporting [48]. In order to mitigate the effects of energy underreporting, the ratio of reported EI to EER (EI:EER) was used to determine the accuracy of reported EI and was included as a covariate in select statistical models [6, 49, 50]. The EER equation used in the Dietary Reference Intakes (DRI) is based on age, sex, weight, height, and physical activity level (PAL) [51]. Low PAL (i.e., 1.4–1.59) was assumed because data from the physical activity questionnaire were not adequate to estimate PAL and physical activity monitor data were not available in the 2013–2014, 2015–2016 cycles of NHANES [31].

### Snacking definitions

Four event-based definitions were operationalized based on energy content, participant definition of a snack, and ingestive events consumed outside of typical meals. These definitions included: (1) any consumption event defined by the reporter as a “snack” or Spanish language equivalent; (2) any consumption event defined by the reporter as a “snack” or Spanish language equivalent that contributed ≥50 kcal; (3) any consumption event outside of a typical meal time (i.e. other than breakfast, lunch, dinner, super, brunch) including self-defined snacks; and (4) any consumption event outside of a typical meal time (i.e. other than breakfast, lunch, dinner, super, brunch) including self-defined snacks that contributed ≥50 kcal. Two of the four definitions took into account a 50 kcal cut-point in order to evaluate each definition with and without occasions that may not be considered an ingestive episode, as some previous studies consider ingestive episodes with less than 50 kcal to be trivial [6]. While it is possible that this method may eliminate low-energy eating occasions (i.e., ingestive episodes containing fruit, vegetables, diet beverages, and/or other reduced-energy snacks), and in turn, attenuate the relationship between snacking frequency and weight status; a sensitivity analysis using a 5 kcal cut-point definition, as conducted in previous work [16, 17], did not differ from a 50 kcal cut-point. The inclusion of two additional snack definitions based on percentage of total daily energy were explored because of its use differentiating meals in other epidemiological trials investigating meal pattern behaviors [7]. These definitions included any consumption event that contributed < 15% of total daily energy intake and any consumption event that contributed < 15% of total daily energy intake and contributed ≥50 kcal. However, due to the fact that breakfast meals typically contribute approximately 18% of total daily energy among adult men and women [52], this definition likely misclassifies breakfast meals as snacks. Therefore, this definition lacks specificity and is not appropriate for this analysis. (Table 1).

**Table 1:** Snacking definitions.

Abbreviation	Snacking Definition
**1. Snacks**	Any event defined by the reporter as a “snack”
**2. Snacks, ≥50 kcal**	Any event defined by the reporter as a “snack” that contributed **≥50 kcal**
**3. Snacks + other eating between meals**^1^	Any event outside of a typical meal time (i.e. other than breakfast, lunch, dinner, super, brunch)
**4. Snacks + other eating between meals, ≥50 kcal**^1^	Any event outside of a typical meal time (i.e. other than breakfast, lunch, dinner, super, brunch) that contributed **≥50 kcal**

^1^ These definitions include both eating and drinking events that meet these criteria. Described as eating for the purposes of simplicity.

### Anthropometric characteristics

Three indicators of weight status were chosen for examination in this analysis, including BMI, WC, and SAD. BMI, obtained from height and weight collected by trained health technicians during the health examination in the MEC, was calculated as weight in kg divided by height in meters squared. Standard BMI categories were constructed: underweight (<18.5, excluded), normal (18.5–24.9), overweight (25.0–29.9), and obese (≥30) [53]. Overweight and obese (OW/OB) were combined because few demographic differences were observed with exceptions noted for race/Hispanic origin in men and women, and PIR in women. In addition to BMI, WC and SAD were also collected during the health examination by trained health technicians using standardized NHANES protocol [54]. WC cut-points of ≥102 cm for men and ≥88 cm for women were used to define abdominal obesity, as well as SAD cut-points of ≥25 cm and ≥24cm in men and women, respectively [29, 30, 55].

### Statistical analysis

Statistical analyses were performed using SAS software (version 9.4; SAS Institute Inc., Cary, NC) and SAS-callable SUDAAN (version 11; Research Triangle Institute, Raleigh, NC, USA) software programs to account for differential nonresponse and non-coverage, and to adjust for oversampling and post-stratification by use of the NHANES dietary day 1 sample weights. PROC DESCRIPT was used to compute prevalence estimates and means of a group for demographic characteristics; a *p*-value of <0.01 was considered statistically significant (Table 2, S1 Table). Associations between BMI and mean snacking frequency, mean energy of a snack event, and percent daily energy from snacks adjusted for EI:EER were investigated for each of the snacking definitions using multiple linear regression analysis derived from the PROC REGRESS procedure (Table 3). Satterthwaite-adjusted F-tests were conducted to assess differences in mean number of snacks per day, mean energy of a snack event, and percent daily energy from snacks for each of the snacking definitions across BMI categories when compared to those with a normal weight, respectively, at a two-sided *p*-value of <0.001 (Table 3). Logistic regression (PROC RLOGIST) was used to evaluate the odds ratio (OR) of overweight and obesity (OW/OB, BMI ≥ 24.9 kg/m^2^), elevated WC risk (WC ≥102 cm men; ≥88 cm women), and elevated SAD risk (SAD ≥25 cm; ≥24 cm), relative to snacking frequency when compared to the referent group of 0 snacks per day (i.e., the lowest snack category) (Tables 4 and 5). Because of the large number of snacking definitions and models examined and the exploratory nature of this analysis, a p value of < 0.001 was established to minimize the chance of a Type I error. A Taylor Series Linearization approach was used to approximate standard errors (SEs) for all estimates.

**Table 2:** Demographic characteristics of study population, NHANES 2013–2016^1^^,^^2^.

	Total	Men	Women
	% (SE)	% (SE)	% (SE)
	*n* = 9,633	*n* = 4,726	*n* = 4,907
**Age in years, *mean (SE)***	48.3 (0.4)	47.2 (0.4)	49.3 (0.5)^†^
**Race/Hispanic Origin**			
**Non-Hispanic White**	65.3 (2.4)	65.7 (2.4)	64.9 (2.5)
**Non-Hispanic Black**	11.1 (1.3)	10.5 (1.2)	11.7 (1.4)
**Hispanic**	14.9 (1.8)	15.0 (1.7)	14.9 (1.8)
**Non-Hispanic Asian**	5.5 (0.8)	5.3 (0.8)	5.6 (0.8)
**Other**	3.2 (0.3)	3.5 (0.5)	3.0 (0.4)
**BMI (kg/m**^**2**^**), *mean (SE)***	29.5 (0.2)	29.1 (0.2)	29.8 (0.2)^†^
**BMI Category**^3^			
**Normal Weight**	27.4 (0.8)	24.7 (1.0)	30.0 (1.1) ^†^
**Overweight**	33.2 (0.6)	38.6 (1.2)	28.0 (0.8) ^†^
**Obese**	39.3 (1.0)	36.7 (1.4)	41.9 (1.1) ^†^
**Smoking Status**	***n* = 9,623**	***n* = 4,720**	***n* = 4,903**
**Never Smoker**	55.8 (0.8)	49.5 (1.1)	62.0 (1.0) ^†^
**Former Smoker**	25.3 (0.8)	30.4 (1.1)	20.4 (1.0) ^†^
**Current, Occasional Smoker**	4.1 (0.3)	5.1 (0.5)	3.1 (0.3) ^†^
**Current, Daily Smoker**	14.7 (0.6)	15.0 (0.7)	14.5 (0.9)
**PIR**	***n* = 8,823**	***n* = 4,332**	***n* = 4,491**
**PIR ≤ 130%**	22.7 (1.5)	20.7 (1.6)	24.7 (1.6) ^†^
**130% < PIR ≤ 350%**	35.4 (1.0)	35.2 (1.2)	35.7 (1.0)
**PIR > 350%**	41.8 (1.9)	44.1 (2.1)	39.6 (1.9) ^†^

Abbreviations: BMI, body mass index (calculation as weight in kilograms divided by height in meters squared); SE, standard error; PIR, family income-to-poverty ratio.

^1^ Values are percent (SE) unless otherwise indicated.

^2^ A dagger “†” denotes significant differences within a row (i.e., sex groups) at *p*-value of < 0.01.

^3^ BMI Categories (kg/m^2^): Normal weight, 18.5≤ BMI <25; Overweight, 25≤ BMI <30; Obese, BMI ≥ 30. Underweight (BMI < 18.5, n = 144) were excluded due to a small sample size.

**Table 3:** Snacking frequency and energy contributed by snack by Body Mass Index (BMI) category among U.S. adults (≥20y), NHANES 2013–2016^1^^-^^4^.

	Men				Women			
	Normal (N = 1243)	Overweight (N = 1807)	Obese (N = 1676)	Total (N = 4726)	Normal (N = 1356)	Overweight (N = 1348)	Obese (N = 2203)	Total (N = 4907)
**Mean number of snacks per day (times/day), *mean (SE)***								
Snacks^5^	1.4 (0.07)	1.4 (0.05)	1.5 (0.06)	1.4 (0.04)	1.7 (0.06)	1.6 (0.05)	1.6 (0.05)	1.6 (0.03)
Snacks, ≥50 kcal^5^^,^*	1.2 (0.06)	1.2 (0.05)	1.2 (0.05)	1.2 (0.04)	1.3 (0.05)	1.3 (0.04)	1.3 (0.04)	1.3 (0.03)
Snacks + other eating between meals^6^	2.8 (0.09)	2.9 (0.08)	2.9 (0.09)	2.9 (0.06)	3.1 (0.09)	2.9 (0.06)	3.0 (0.07)	3.0 (0.05)
Snacks + other eating between meals, ≥50 kcal^6^^,^*	1.7 (0.07)	1.8 (0.05)	1.8 (0.06)	1.8 (0.04)	1.8 (0.06)	1.7 (0.04)	1.8 (0.04)	1.7 (0.03)
**Mean energy of a snack event (kcal/snack), *mean (SE)***								
Snacks^5^	59.6 (4.44)	66.8 (2.99)	73.3 (2.52)	67.4 (1.64)	48.1 (1.89)	54.2 (2.06)	57.4 (1.92)	53.7 (1.09)
Snacks, ≥50 kcal^5^^,^*	72.2 (5.75)	81.3 (3.57)	88.6 (3.17)	81.8 (2.11)	60.4 (2.50)	68.6 (2.81)	70.3 (2.54)	66.8 (1.56)
Snacks + other eating between meals^6^	101.8 (5.28)	108.1 (4.37)	124.1(3.29)	112.4 (2.27)	69.0 (2.79)	77.8 (3.12)	85.5 (3.02)^†^	78.3 (1.85)
Snacks + other eating between meals, ≥50 kcal^6^^,^*	120.0 (5.79)	130.4 (4.99)	147.2 (4.32)	134.0 (2.75)	85.3 (3.50)	95.9 (3.80)	103.3(3.97)^†^	95.8 (2.49)
**Percent of daily energy from snacks (%), *mean (SE)***								
Snacks^5^	15.2 (0.75)	15.8 (0.65)	15.3 (0.71)	15.5 (0.42)	17.2 (0.73)	18.0 (0.66)	16.9 (0.59)	17.3 (0.40)
Snacks, ≥50 kcal^5^^,^*	15.0 (0.74)	15.6 (0.65)	15.2 (0.72)	15.3 (0.42)	16.7 (0.72)	17.6 (0.67)	16.6 (0.60)	16.9 (0.40)
Snacks + other eating between meals^6^	23.9 (0.73)	24.2 (0.89)	24.3 (0.75)	24.2 (0.54)	24.1 (0.94)	25.0 (0.89)	24.0 (0.76)	24.3 (0.54)
Snacks + other eating between meals, ≥50 kcal^6^^,^*	23.6 (0.72)	23.9 (0.89)	24.0 (0.75)	23.9 (0.54)	23.4 (0.93)	24.3 (0.89)	23.5 (0.77)	23.7 (0.53)

^1^ Values are mean (SE) unless otherwise indicated. All estimates were calculated using multiple linear regression adjusted for EI:EER.

^2^ A dagger “†” denotes a significant difference in the mean number of snacks per day or mean energy of a snack event when compared with the mean number of snacks per day or mean energy of a snack event of the referent group (i.e., normal weight), respectively, within sex groups. A *p*-value of <0.001 was considered statistically significant.

^3^ BMI Categories (kg/m^2^): Normal weight, 18.5≤ BMI <25; Overweight, 25≤ BMI <30; Obese, BMI ≥ 30. Underweight (BMI < 18.5, n = 144) were excluded due to a small sample size.

^4^ An asterisk “*”denotes a snack defined as an event that contributed ≥ 50 kcal.

^5^ Snack defined as an event defined by the reporter as a “snack.”

^6^ Snack defined as an event outside of a typical mealtime. These definitions include both eating and drinking events that meet these criteria. Described as eating for the purposes of simplicity.

**Table 4:** Odds ratio of overweight or obesity (BMI ≥ 25 kg/m^2^), Waist Circumference (WC) >102cm, and Sagittal Abdominal Diameter (SAD) >25cm with snacking frequency among U.S. adult men (≥20y), NHANES 2013–2016^1^^-^^3^.

		BMI		WC		SAD	
Daily Occasions	n	OR (95% CI)	P	OR (95% CI)	P	OR (95% CI)	P
**Snacks**^4^							
0	1335	1.00 (Ref)	-	1.00 (Ref)	-	1.00 (Ref)	-
1	1534	1.04 (0.79, 1.36)	0.78	0.99 (0.71, 1.38)	0.93	1.14 (0.76, 1.69)	0.51
2	1083	0.92 (0.67, 1.28)	0.62	0.70 (0.49, 1.00)	0.05	0.98 (0.61, 1.56)	0.92
3	490	0.90 (0.61, 1.32)	0.58	0.78 (0.55, 1.12)	0.17	1.06 (0.64, 1.76)	0.80
4+	284	0.93 (0.50, 1.71)	0.81	1.01 (0.55, 1.83)	0.98	1.18 (0.63, 2.23)	0.59
**Snacks, ≥ 50 kcal occasions**^4^^,^*							
0	1567	1.00 (Ref)	-	1.00 (Ref)	-	1.00 (Ref)	-
1	1619	1.03 (0.78, 1.35)	0.84	0.90 (0.67, 1.21)	0.49	1.04 (0.74, 1.46)	0.82
2	1011	0.95 (0.65, 1.39)	0.79	0.63 (0.43, 0.91)	0.01	0.91 (0.57, 1.45)	0.68
3	361	0.90 (0.58, 1.42)	0.65	0.82 (0.56, 1.19)	0.28	1.09 (0.68, 1.75)	0.72
4+	168	1.01 (0.47, 2.14)	0.99	0.90 (0.41, 1.97)	0.79	1.03 (0.51, 2.11)	0.92
**Snacks + other eating between meals**^5^							
0	419	1.00 (Ref)	-	1.00 (Ref)	-	1.00 (Ref)	-
1	931	1.09 (0.75, 1.59)	0.64	0.87 (0.63, 1.21)	0.40	1.02 (0.59, 1.77)	0.94
2	1080	0.95 (0.60, 1.50)	0.81	0.81 (0.57, 1.15)	0.23	1.03 (0.58, 1.83)	0.91
3	958	1.23 (0.78, 1.92)	0.36	1.04 (0.71, 1.53)	0.82	1.35 (0.75, 2.44)	0.31
4+	1338	1.12 (0.72, 1.76)	0.61	0.86 (0.57, 1.29)	0.45	1.02 (0.54, 1.93)	0.94
**Snacks + other eating between meals, ≥50 kcal occasions**^5^^,^*							
0	999	1.00 (Ref)	-	1.00 (Ref)	-	1.00 (Ref)	-
1	1386	1.25 (1.01, 1.55)	0.04	0.85 (0.65, 1.12)	0.24	1.05 (0.75, 1.48)	0.77
2	1214	1.16 (0.84, 1.59)	0.36	0.76 (0.54, 1.09)	0.13	1.13 (0.78, 1.62)	0.51
3	683	1.18 (0.76, 1.83)	0.45	0.65 (0.41, 1.03)	0.06	1.01 (0.63, 1.61)	0.97
4+	444	1.28 (0.80, 2.02)	0.29	0.91 (0.56, 1.49)	0.71	1.14 (0.66, 1.96)	0.64

Abbreviations: Ref, Reference Value.

^1^ OW/OB: Odds ratio of overweight or obesity (BMI≥25); WC: Odds ratio of waist circumference > 102cm; SAD: Odds ratio of SAD>25cm; EER: Men EER = 662 –(9.53 x age [y]) + PA x [(15.91 x weight [kg]) + (539.6 x height [m])], Women EER = 354 –(6.91 x age [y]) + PA x [(9.36 x weight [kg]) + (726 x height [m])]. A *p*-value of < 0.001 was considered statistically significant.

^2^ An asterisk “*”denotes a snack defined as an event that contributed ≥ 50 kcal.

^3^ Covariates included in the model (Model 1): age (continuous), race (NH—White, NH-Black, NH-Asian, Hispanic), family income-to-poverty ratio (PIR ≤ 130%, 130% < PIR ≥ 350%, PIR > 350%), day of recall (weekend, weekday), typical intake (usual intake, much more than usual, much less than usual), smoking status (never smoked, former smoker, current smoker occasionally, current smoker daily), EI:EER, mean energy of a snack, and mean energy density (kcal/g) of a snack.

^4^ Snack defined as an event defined by the reporter as a “snack.”

^5^ Snack defined as an event outside of a typical mealtime.

**Table 5:** Odds ratio of overweight or obesity (BMI ≥ 25 kg/m^2^), Waist Circumference (WC) >102cm, and Sagittal Abdominal Diameter (SAD) >25cm with snacking frequency among U.S. adult women (≥20y), NHANES 2013–2016^1^^-^^3^.

		BMI		WC		SAD	
Daily Occasions	n	OR (95% CI)	P	OR (95% CI)	P	OR (95% CI)	P
**Snacks**^4^							
0	1047	1.00 (Ref)	-	1.00 (Ref)	-	1.00 (Ref)	-
1	1617	1.03 (0.71, 1.50)	0.85	1.20 (0.86, 1.67)	0.28	1.09 (0.86, 1.39)	0.46
2	1305	0.88 (0.55, 1.40)	0.57	1.00 (0.68, 1.47)	0.99	1.08 (0.82, 1.41)	0.58
3	609	0.76 (0.45, 1.28)	0.29	0.94 (0.63, 1.38)	0.73	0.96 (0.63, 1.48)	0.85
4+	329	0.73 (0.42, 1.27)	0.26	0.92 (0.53, 1.57)	0.74	1.53 (1.05, 2.21)	0.03
**Snacks, ≥50 kcal occasions**^4^^,^*							
0	1289	1.00 (Ref)	-	1.00 (Ref)	-	1.00 (Ref)	-
1	1889	0.91 (0.67, 1.23)	0.53	0.94 (0.69, 1.29)	0.70	1.11 (0.83, 1.48)	0.47
2	1160	0.77 (0.48, 1.23)	0.27	0.88 (0.58, 1.33)	0.52	1.05 (0.75, 1.49)	0.76
3	418	0.72 (0.45, 1.16)	0.17	0.92 (0.54, 1.56)	0.74	1.35 (0.83, 2.20)	0.22
4+	151	0.63 (0.35, 1.15)	0.13	0.58 (0.31, 1.08)	0.08	2.05 (1.08, 3.89)	0.03
**Snacks + other eating between meals**^5^							
0	285	1.00 (Ref)	-	1.00 (Ref)	-	1.00 (Ref)	-
1	865	0.70 (0.38, 1.29)	0.24	0.82 (0.41, 1.64)	0.57	0.74 (0.40, 1.36)	0.32
2	1185	0.79 (0.46, 1.36)	0.39	1.09 (0.60, 1.97)	0.76	0.92 (0.59, 1.44)	0.70
3	1081	0.88 (0.51, 1.52)	0.64	0.93 (0.50, 1.72)	0.81	0.69 (0.43, 1.10)	0.11
4+	1491	0.70 (0.38, 1.29)	0.24	0.94 (0.49, 1.80)	0.84	0.86 (0.52, 1.43)	0.56
**Snacks + other eating between meals, ≥50 kcal occasions**^5^^,^*							
0	878	1.00 (Ref)	-	1.00 (Ref)	-	1.00 (Ref)	-
1	1599	0.69 (0.51, 0.95)	0.02	0.77 (0.59, 1.00)	0.05	1.02 (0.78, 1.35)	0.86
2	1346	0.75 (0.47, 1.20)	0.22	0.77 (0.51, 1.16)	0.21	0.75 (0.54, 1.06)	0.10
3	703	0.70 (0.42, 1.18)	0.18	0.87 (0.55, 1.37)	0.53	1.05 (0.68, 1.62)	0.81
4+	381	0.58 (0.34, 0.98)	0.04	0.69 (0.43, 1.10)	0.11	1.39 (0.89, 2.17)	0.14

Abbreviations: Ref, Reference Value.

^1^ OW/OB: Odds ratio of overweight or obesity (BMI≥25); WC: Odds ratio of waist circumference > 102cm; SAD: Odds ratio of SAD>25cm; EER: Men EER = 662 –(9.53 x age [y]) + PA x [(15.91 x weight [kg]) + (539.6 x height [m])], Women EER = 354 –(6.91 x age [y]) + PA x [(9.36 x weight [kg]) + (726 x height [m])]. A *p*-value of < 0.001 was considered statistically significant.

^2^ An asterisk “*”denotes a snack defined as an event that contributed ≥ 50 kcal.

^3^ Covariates included in the model (Model 1): age (continuous), race (NH—White, NH-Black, NH-Asian, Hispanic), family income-to-poverty ratio (PIR ≤ 130%, 130% < PIR ≥ 350%, PIR > 350%), day of recall (weekend, weekday), typical intake (usual intake, much more than usual, much less than usual), smoking status (never smoked, former smoker, current smoker occasionally, current smoker daily), EI:EER, mean energy of a snack, and mean energy density (kcal/g) of a snack.

^4^ Snack defined as an event defined by the reporter as a “snack.”

^5^ Snack defined as an event outside of a typical mealtime.

Several models comparing snacking frequency by definition with weight status were examined. First, we examined the crude, unadjusted relationship between snacking and weight status. Second, we built models including factors related to the exposure and outcome and derived fully adjusted models, including demographic, lifestyle, and dietary factors related to the research question and informed by previous analyses, as presented in the main tables [6, 16, 17, 46]. In supplemental tables, we present additional models that are more parsimonious as part of a sensitivity analysis to examine the effects of covariate selection.

## Results

As representative of the adult U.S. population, this analytic sample was approximately 48.3 years of age, non-Hispanic white (65.3%), primarily women (50.7%), and had overweight (33.2%) or obesity (39.3%) (Table 2). While more men were classified as overweight, more women were classified as obese. Overall, the majority of adults did not have a history of smoking (56.1%), but sex differences were noted. Most Americans fall in to the low or middle PIR categories. Additional details regarding the demographic characteristics of men and women included in our analytic sample by weight status are provided in S1 Table.

**Fig 1:**
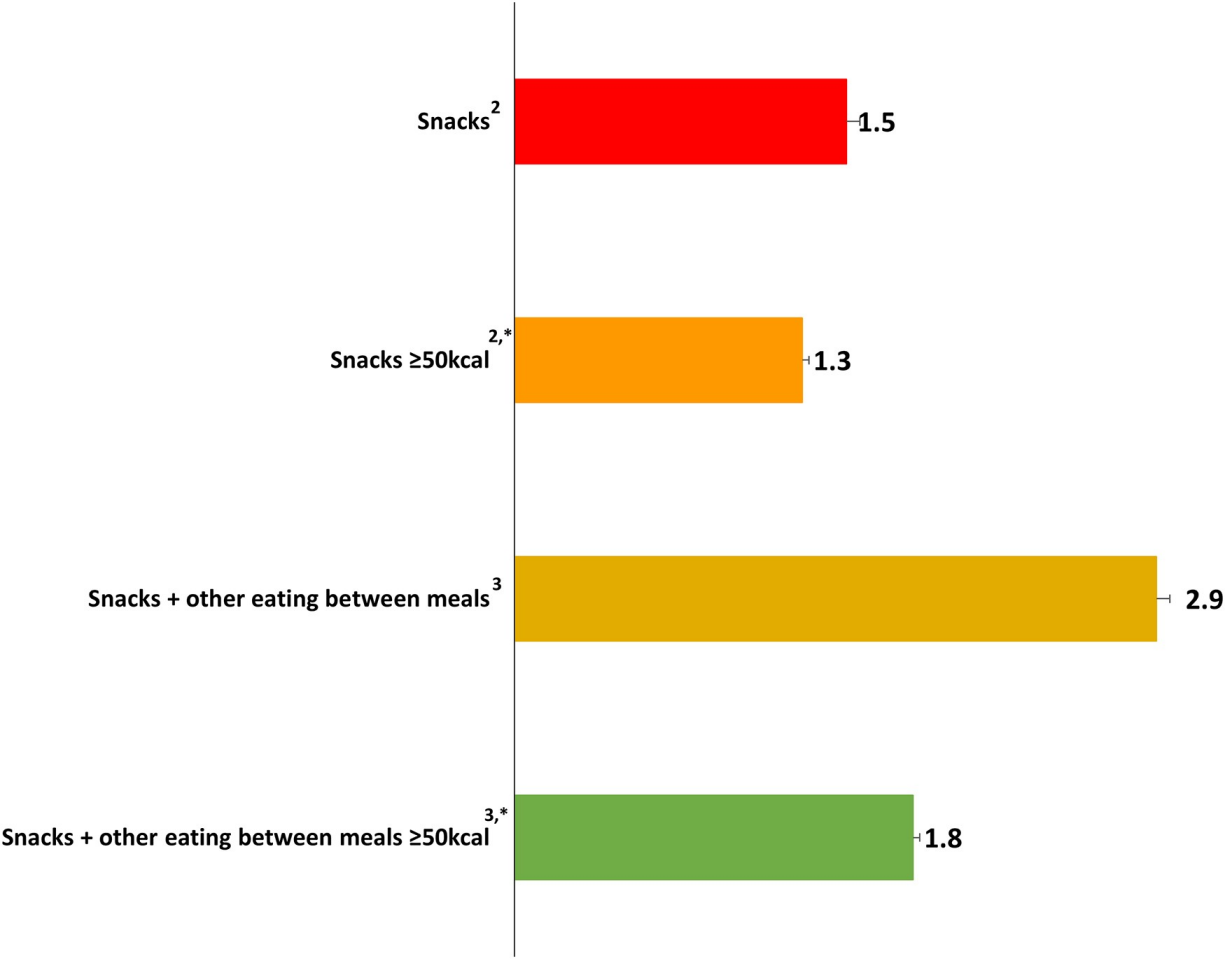
Mean number of snacks per day by snack definition among U.S. Adults (≥20y), NHANES 2013−2016^1^. ^**1**^ Values are means unless otherwise indicated. An asterisk “*”denotes a snack defined as an event that contributed ≥ 50 kcal. ^2^ Snack defined as an event defined by the reporter as a “snack.” ^3^Snack defined as an event outside of a typical mealtime.

The number of daily snacking occasions varied in magnitude by the snack definition employed, and generally ranged between 1.2 and 3.0 snacks per day among men and women (Table 3). The frequency of snack consumption was highest when a snack was defined as any eating occasion outside of a typical meal time (Snacks + other eating between meals), with this definition yielding an average of approximately three snacks per day (Fig 1). Frequency of snacking was generally similar between men and women across the various snack definitions, with the biggest difference in snack frequency being when a snack was defined by the participant (i.e. Snacks). No significant differences in number of snacks consumed were observed when comparing weight status categories, regardless of the snack definition among men and women.

Mean snack energy ranged from 67.4 to 134.0 kcal per snacking occasion among men, and from 54.1 to 96.2 kcal per snacking occasion among women, contributing approximately 15.3–24.2% and 16.9–24.3% of total daily energy among men and women, respectively (Table 3). Snacks defined as any event outside of a typical meal time that contributed more than 50 kcal were consistently higher in snack energy content, as opposed to snacks defined by the participant, which were the lowest in snack energy content out of the four snacking definitions operationalized. Mean energy from snacks differed by weight status in women but not men. Specifically, when snacks were defined as any event outside of a typical meal time, mean energy from snacks was significantly higher (~15–20 kcals/snack) among women with obesity compared to women with normal weight, regardless of whether it contributed more than 50 kcal (Snacks + other eating between meals and Snacks + other eating between meals, ≥50 kcal). These findings were not congruent with percent of daily energy from snacks; unlike mean snack energy, the percent of daily energy contributed by snacks did not differ by weight status among men and women, but varied by snack definition (Table 3). Broadly, snacks defined as any event outside of a typical meal time provided approximately 24% of total daily energy, regardless of whether it contributed more than 50 kcal (Snacks + other eating between meals and Snacks + other eating between meals, ≥50 kcal), as opposed to snacks defined by the participant, which contributed only 15–17% of total daily energy, regardless of whether the snack contributed more than 50 kcal (Snacks and Snacks, ≥50 kcal). Yet, these differences did not differ substantially by weight status categories.

In general, the relationship between snacking frequency and adiposity was largely null across all indicators of weight status (i.e., OW/OB, WC, and SAD) (Tables 4 and 5). In both men and women, frequency of snacking was not significantly associated with adverse weight status for any of the definitions examined (Tables 4 and 5); yet, these associations did vary somewhat by indicator of weight status. Most notably, men who consumed two snacks per day tended (p = 0.01) to be at lower risk of elevated WC than men who did not consume any snacks (i.e., 0 snacks per day), when a snack was defined by the participant and contributed more than 50 kcal; however, this difference did not reach statistical significance, and this association was not observed with increased risk of OW/OB or elevated SAD. Furthermore, even when evaluating the relationship between weight status indicators and snacking frequency in further models with more minimal adjustments, no associations were observed among men or women when adjusting for fewer covariates than those included in the fully adjusted models (S2 and S3 Tables).

## Discussion

Overweight and obesity persist as significant public health issues in the U.S. Understanding modifiable factors that are related to unhealthy weight status in adults is critical for the development of public health interventions aimed at reducing the risk of overweight, obesity, and their related comorbidities. Snacking is one behavior that has been implicated in the etiology of overweight/obesity. Snacking incidence has increased markedly over the past four decades [2] and it is estimated to contribute approximately 20–25% of daily energy [1, 56, 57]. In North America, Northern Europe, and South America, snacking contributes approximately as much energy as breakfast to the daily diet [56]. While snacking and eating frequency have been associated with positive energy balance, the link to overweight/obesity has been less clearly established [27, 58]. One possible contributor to this apparent lack of congruency may be the inconsistency in definitions of snacking as this could add considerable variance to its estimation. While associations between snack characteristics and adiposity varied by event-based definitions of snacking as hypothesized, the association between snack frequency and adiposity was largely null among U.S. adults.

Two important dimensions of snacking are its possible contribution to eating frequency and the energy content of snacking events. This analysis focused on the effects of frequency and size of snacking occasions on multiple metrics of adiposity in a nationally representative sample of U.S. adults. While BMI categories and WC are primarily used to define weight status, SAD is an additional proposed measure that may provide a more accurate indicator of adiposity associated with increased risk of adverse metabolic effects/metabolic syndrome [23, 29, 30].

This analysis indicated snack frequency was not associated with weight status regardless of the event-based definition tested. However, snack definition did play a role in the associations between weight status, mean energy from snacks among women, and the percent of total daily energy from snacks. Most notably, mean energy from snacks among women with obesity was higher than in women with a normal weight when a snack was defined as any ingestive event between meals (Snacks + other eating between meals), but not when a snack was defined by the participant (Snacks). This difference in mean energy between definitions suggests that energy from other ingestive events between typical meal times that participants do not consider to be snacks may be underestimated or poorly compensated and contribute to excess energy intake. Snack frequency and percent of total energy from snacks were not significantly different between women with a normal weight and women with obesity. Because this association is not statistically significant in men, other psychosocial factors associated with sex may play a role, or may be related to the omission of reported snacks by men [59, 60]. Total percent daily energy from snacks was different between snack definitions, which is attributed to potentially more ingestive occasions being included in the snack definitions that included all events outside of typical meal times (Snacks + other eating between meals). This further emphasizes that events consumed outside of typical meal times may not be considered snacks by the consumer. This is not just a semantic issue; these events that are not defined by the consumer as snacks or meals potentially contribute as much as 7–9% of total energy intake (17–19% of energy with Snacks definition versus 24% of energy with Snacks + other eating between meals), or approximately 140 to 180 kcal assuming a 2,000 kcal diet. Therefore, measurement of snacks defined by the consumer and typical meal times may underestimate total daily energy intake. Despite the potential ramifications the different definitions may have on interpretation of research findings, there was generally no difference in adiposity risk between the different snack definitions utilized in this analysis.

The second dimension of snacking contributes to total energy intake and is related to the energy content of snacking events, which was not investigated in this analysis. An investigation into the association between total snack energy intake and weight status may provide insight into whether snack energy intake opposed to snack frequency promotes differences in weight status. It would also be useful to examine food form or snacks as beverages, which may evoke weaker energy compensation [61] than solid foods and are common components of snacking occasions [62, 63].

Each event-based definition included in the analysis addresses a slightly different question regarding the association between snack frequency and weight status. Defining a snack based on the participant’s self-report is culturally relevant and provides insight into how snacks are defined by the general population. However, each subject has their own individual classification system to define their ingestive events that could be based on a variety of criteria including time of day, type of food consumed, or portion size. This variability in snack classification between subjects introduces error leading to an over- or under-estimation of snack frequency. In addition, sugar-sweetened beverages, which can contribute substantially (236–388 kcal/day among U.S. adults [64]) to total energy intake [65], are commonly not classified as snacks or not reported entirely despite meeting the criteria of a snack (e.g., consumption between meals and energy content lower than typical meals). Defining a snack based on events consumed outside of typical meal times does provide a more systematic measure of snack frequency than self-report. However, because this definition does not take into account energy content, events consumed outside typical meals that may be equivalent to the energy consumed within a meal could in fact contribute substantially to total daily energy intake. Therefore, results should be interpreted in the context of the snack definition utilized and the question each definition addresses in this and other analyses.

At this time, an association between snacking and weight status among adults continues to be debated, and evidence remains mixed. Murakami and colleagues [6] observed models adjusted for the accuracy of energy reporting resulted in a positive or null association between snacking frequency and risk of OW/OB in both men and women. Notably, the odds of OW/OB was associated with snacking frequency when snacking was defined as a “snack” by the reporter or by percent of total daily energy intake among men, and when snacking was defined by percent of total daily energy intake (but not when defined by the reporter) among women [6]. However, the cut off used by Murakami et al. to define a meal based on percent of total daily energy intake was 15% of total daily energy [6], which may misclassify breakfast meals due to the low energy content of breakfast compared to other meal times (i.e., ~18% of total daily energy among U.S. men and women [52]). In addition, models employed in this analysis controlled for protein, fat, total sugar, alcohol, and fiber intake, which may over adjust or confound the model, biasing resultant findings [66]. The results of our current analysis do not support previous literature, which may be related to the inclusion of snack energy density in the model.

These largely null associations also differ from our previous work in children. A recent study conducted by Tripicchio et al. among U.S. adolescents concluded that adolescents with overweight and obesity not only consumed a greater number of snacks per day than their normal weight counterparts, but also have a higher energy intake at each snacking occasion than those with a normal weight [17]. These findings by Tripicchio et al. are concordant with research conducted by Kachurak et al. in children, specifically among those under 2 years of age, where positive associations between weight status and snacking were consistently seen when the snacking definitions accounted for ingestive events outside of traditional meal time [16]. Kachurak et al. also determined that regardless of age, children with overweight and obesity were more likely to have greater daily energy intake than their normal weight counterparts, which may, in turn, corroborate their findings [16]. Growth is a time of increasing energy need, so the relationship with snacking observed in children does not appear to be similar to our findings in adults.

### Strengths and limitations

NHANES is a nationally representative survey of the U.S. noninstitutionalized population. This analysis was designed to provide a framework to evaluate the association between snacking and body weight status at the population-level. The USDA’s AMPM is a state of the art method for dietary data collection as is the FNDDS database used to process dietary data. However, self-reported dietary data are prone to systematic errors, and tend to underestimate true energy intake [67]. Nevertheless, there are many important uses of self-reported dietary data, especially for monitoring trends over time for nutrition and health outcomes [48]. In order to reduce potential bias in energy underreporting, we included the EI:EER ratio in our models to account for reporting bias to the extent possible. Because detailed information on physical activity was not available in these survey years, a low physical activity was assumed based on prior research suggesting this is a reasonable assumption based on previous research that included activity monitors [6, 68, 69]. However, assuming all individuals are sedentary or participate in low physical activity would bias estimates for adults who report moderate or vigorous physical activity. In addition to systemic underestimation of total energy intake, snack foods are more likely to be underreported compared to foods consumed as meals [70]. The event-based snack definitions utilized in the analysis are representative of definitions in the published literature; however, other snack definitions such as definitions based on the type of food consumed, amount of energy consumed, or time of day may provide additional insight into the relationship between snack frequency and adiposity and warrant further exploration. Our findings are limited by the cross-sectional nature of NHANES data, and a causal relationship cannot be definitively determined. We chose to use NHANES to examine this research question, because NHANES is a nationally representative sample of the US population and allowed for analysis of snacking patterns at the population level. Longitudinal cohort studies and randomized controlled trials are needed to address the causal relationship, if any, between frequency and definition of snacking with weight status. Due to the exploratory nature of this analysis, a p value of < 0.001 was established to minimize the chance of a Type I error; however, under a less restrictive p value of <0.05, some significant findings were observed, suggesting that the conservative approach taken may serve as a potential limitation of our analysis. OW/OB results from a constellation of dietary, genetic, behavioral, and environmental factors and remains of serious public health concern given that OW/OB increases the risk of metabolic syndrome, cardiovascular disease, type 2 diabetes mellitus, and some cancers [71–73]. Furthermore, while we made every attempt to control for known confounding variables, given the cross-sectional nature of this study and the complexity of etiologic factors that are related to weight, we cannot rule out the potential of residual confounding.

## Conclusions

Despite hypotheses that the use of different event-based definitions of snacking may account for the mixed findings on the role of snacking in weight management, this analysis did not yield robust effects of the definitions tested. Confirmation of these findings based on cross-sectional survey data with longitudinal assessments and clinical trials is warranted given the high level of energy contributed by snacks and consequent potential challenge they pose to achieving a healthy weight.

## Supporting information

S1 Text List of abbreviations. (DOCX)

S1 Table Demographic characteristics by weight status, NHANES 2013–2016. ^**1–4**^ Abbreviations: BMI, body mass index (calculation as weight in kilograms divided by height in meters squared); SE, standard error; PIR, family income-to-poverty ratio. ^1^ Values are percent (SE) unless otherwise indicated. ^2^ Different superscript letters (a,b,c) indicate significant differences within a row (i.e., weight status categories) at *p*-value of < 0.01. ^3^ BMI Categories (kg/m^2^): Normal weight, 18.5≤ BMI <25; Overweight, 25≤ BMI <30; Obese, BMI ≥ 30. Underweight (BMI < 18.5, n = 144) were excluded due to a small sample size. ^4^ Race/Hispanic Origin does not sum to 100 because the other race category is not shown.
(DOCX)

S2 Table Odds ratio of overweight or obesity (BMI ≥ 25 kg/m^2^), Waist Circumference (WC) >102cm, and Sagittal Abdominal Diameter (SAD) >25cm with snacking frequency among U.S. adult men (≥20y) in models controlling for additional covariates, NHANES 2013–2016. ^**1–2**^ Abbreviations: Ref, Reference Value. ^1^ OW/OB: Odds ratio of overweight or obesity (BMI≥25); WC: Odds ratio of waist circumference > 102cm; SAD: Odds ratio of SAD>25cm; EER: Men EER = 662 –(9.53 x age [y]) + PA x [(15.91 x weight [kg]) + (539.6 x height [m])], Women EER = 354 –(6.91 x age [y]) + PA x [(9.36 x weight [kg]) + (726 x height [m])]. A *p*-value of < 0.001 was considered statistically significant. ^2^ An asterisk “*”denotes a snack defined as an event that contributed ≥ 50 kcal. ^3^ Snack defined as an event defined by the reporter as a “snack.” ^4^ Covariates included in Model 2: age (continuous), race (NH—White, NH-Black, NH-Asian, Hispanic), family income-to-poverty ratio (PIR ≤ 130%, 130% < PIR ≥ 350%, PIR > 350%), day of recall (weekend, weekday), typical intake (usual intake, much more than usual, much less than usual), smoking status (never smoked, former smoker, current smoker occasionally, current smoker daily), and EI:EER. ^5^ Covariates included in Model 3: covariates included in Model 2 with the exception of EI:EER, and the addition of mean energy of a snack and mean energy density (kcal/g) of a snack. ^6^ Snack defined as an event outside of a typical mealtime.
(DOCX)

S3 Table Odds ratio of overweight or obesity (BMI ≥ 25 kg/m^2^), Waist Circumference (WC) >102cm, and Sagittal Abdominal Diameter (SAD) >25cm with snacking frequency among U.S. adult women (≥20y) in models controlling for additional covariates, NHANES 2013−2016. ^**1,2**^ Abbreviations: Ref, Reference Value. ^1^ OW/OB: Odds ratio of overweight or obesity (BMI≥25); WC: Odds ratio of waist circumference > 102cm; SAD: Odds ratio of SAD>25cm; EER: Men EER = 662 –(9.53 x age [y]) + PA x [(15.91 x weight [kg]) + (539.6 x height [m])], Women EER = 354 –(6.91 x age [y]) + PA x [(9.36 x weight [kg]) + (726 x height [m])]. A *p*-value of < 0.001 was considered statistically significant. ^2^ An asterisk “*”denotes a snack defined as an event that contributed ≥ 50 kcal. ^3^ Snack defined as an event defined by the reporter as a “snack.” ^4^ Covariates included in Model 2: age (continuous), race (NH—White, NH-Black, NH-Asian, Hispanic), family income-to-poverty ratio (PIR ≤ 130%, 130% < PIR ≥ 350%, PIR > 350%), day of recall (weekend, weekday), typical intake (usual intake, much more than usual, much less than usual), smoking status (never smoked, former smoker, current smoker occasionally, current smoker daily), and EI:EER. ^5^ Covariates included in Model 3: covariates included in Model 2 with the exception of EI:EER, and the addition of mean energy of a snack and mean energy density (kcal/g) of a snack. ^6^ Snack defined as an event outside of a typical mealtime.
(DOCX)
