# Supplementary material for: Examination of different definitions of snacking frequency and associations with weight status among U.S. adults
Source: PLoS One. 2020 Jun 17;15(6):e0234355. doi: 10.1371/journal.pone.0234355 (PMC7299329; doi:10.1371/journal.pone.0234355)
Supplement: S1 Text — (DOCX) [file pone.0234355.s001.docx]

**List of Abbreviations**

U.S. United States

NHANES National Health and Nutrition Examination Survey

BMI Body Mass Index

OW/OB Overweight and obesity

WC Waist circumference

SAD Sagittal abdominal diameter

NCHS National Center for Health Statistics

MEC Mobile Examination Center

PIR Family income-to-poverty ratio

24HR 24-hour dietary recall

USDA U.S. Department of Agriculture

FNDDS Food and Nutrient Database for Dietary Studies

EI Energy intake

EER Estimated energy requirement

DRI Dietary Reference Intakes

PAL Physical activity level
