# Supplementary material for: Examination of different definitions of snacking frequency and associations with weight status among U.S. adults
Source: PLoS One. 2020 Jun 17;15(6):e0234355. doi: 10.1371/journal.pone.0234355 (PMC7299329; doi:10.1371/journal.pone.0234355)
Supplement: S1 Table — 1–4 Abbreviations: BMI, body mass index (calculation as weight in kilograms divided by height in meters squared); SE, standard error; PIR, family income-to-poverty ratio. 1 Values are percent (SE) unless otherwise indicated. 2 Different superscript letters (a,b,c) indicate significant differences within a row (i.e., weight status categories) at p-value of < 0.01. 3 BMI Categories (kg/m2): Normal weight, 18.5≤ BMI <25; Overweight, 25≤ BMI <30; Obese, BMI ≥ 30. Underweight (BMI < 18.5, n = 144) were excluded due to a small sample size. 4 Race/Hispanic Origin does not sum to 100 because the other race category is not shown. (DOCX) [file pone.0234355.s002.docx]

**S1 Table.** **Demographic Characteristics by Weight Status, NHANES 2013-2016^1-4^**

|  | **Normal Weight** | **Overweight** | **Obese** |
| --- | --- | --- | --- |
|  | **% (SE)** | **% (SE)** | **% (SE)** |
| **Men** | | | |
| **Age in years, *mean (SE)*** | 43.0 (0.6)^a^ | 48.8 (0.6)^b^ | 48.3 (0.5)^b^ |
| **Race/Hispanic Origin** | ***n* = 1,243** | ***n* = 1,807** | ***n* = 1,676** |
| **Non-Hispanic White** | 61.3 (2.8)^a^ | 68.2 (2.5)^b^ | 66.0 (2.7)^ab^ |
| **Non-Hispanic Black** | 12.8 (1.5)^a^ | 8.7 (1.2)^b^ | 10.7 (1.4)^ab^ |
| **Hispanic** | 11.8 (1.6)^a^ | 15.2 (1.8)^ab^ | 16.9 (2.1)^b^ |
| **Non-Hispanic Asian** | 10.4 (1.8)^a^ | 5.6 (0.9)^b^ | 1.7 (0.2)^c^ |
| **BMI (kg/m^2^), *mean (SE)*** | 22.6 (0.1)^a^ | 27.4 (0.1)^b^ | 35.3 (0.2)^c^ |
| **Smoking Status** | ***n* = 1,242** | ***n* = 1,804** | ***n* = 1,674** |
| **Never Smoker** | 53.5 (2.4) | 48.5 (2.0) | 48.0 (1.9) |
| **Former Smoker** | 21.7 (2.0)^a^ | 32.5 (1.6)^b^ | 34.0 (1.4)^b^ |
| **Current, Occasional Smoker** | 5.3 (1.1) | 4.6 (0.7) | 5.4 (0.9) |
| **Current, Daily Smoker** | 19.5 (2.2) | 14.5 (1.4) | 12.6 (1.0) |
| **PIR** | ***n* = 1,145** | ***n* = 1,643** | ***n* = 1,544** |
| **PIR ≤ 130%** | 25.0 (2.0)^a^ | 18.5 (1.7)^b^ | 20.2 (1.8)^ab^ |
| **130% < PIR ≤ 350%** | 36.9 (2.2) | 34.0 (1.9) | 35.2 (2.0) |
| **PIR > 350%** | 38.1 (3.3)^a^ | 47.5 (2.5)^b^ | 44.5 (2.5)^ab^ |
| **Women** | | | |
| **Age in years, *mean (SE)*** | 47.2 (0.8)^a^ | 50.8 (0.7)^b^ | 49.9 (0.5)^b^ |
| **Race/Hispanic Origin** | ***n* = 1,356** | ***n* = 1,348** | ***n* = 2,203** |
| **Non-Hispanic White** | 68.8 (2.5)^a^ | 66.7 (2.9)^ab^ | 60.9 (3.1)^b^ |
| **Non-Hispanic Black** | 6.6 (1.0)^a^ | 9.5 (1.3)^b^ | 16.8 (2.2)^c^ |
| **Hispanic** | 10.7 (1.5)^a^ | 16.0 (2.2)^b^ | 17.1 (2.2)^b^ |
| **Non-Hispanic Asian** | 11.0 (1.5)^a^ | 4.9 (0.9)^b^ | 2.1 (0.4)^c^ |
| **BMI (kg/m^2^), *mean (SE)*** | 22.2 (0.1)^a^ | 27.4 (0.1)^b^ | 36.8 (0.2)^c^ |
| **Smoking Status** | ***n* = 1,355** | ***n* = 1,346** | ***n* = 2,202** |
| **Never Smoker** | 66.1 (2.2) | 62.1 (1.8) | 59.0 (1.7) |
| **Former Smoker** | 15.9 (1.4)^a^ | 21.2 (1.7)^ab^ | 23.2 (1.6)^b^ |
| **Current, Occasional Smoker** | 3.3 (0.7) | 3.4 (0.7) | 2.8 (0.5) |
| **Current, Daily Smoker** | 14.8 (1.8) | 13.2 (1.4) | 15.0 (1.0) |
| **PIR** | ***n* = 1,248** | ***n* = 1,226** | ***n* = 2,017** |
| **PIR ≤ 130%** | 20.7 (2.1)^a^ | 24.4 (1.8)^ab^ | 27.7 (2.0)^b^ |
| **130% < PIR ≤ 350%** | 31.1 (1.6)^a^ | 35.4 (2.0)^ab^ | 39.1 (1.5)^b^ |
| **PIR > 350%** | 48.2 (2.6)^a^ | 40.2 (2.7)^b^ | 33.2 (2.0)^c^ |

Abbreviations: BMI, body mass index (calculation as weight in kilograms divided by height in meters squared); SE, standard error; PIR,

family income-to-poverty ratio.

^1^ Values are percent (SE) unless otherwise indicated.

^2^ Different superscript letters (a,b,c) indicate significant differences within a row (i.e., weight status categories) at *p*-value of < 0.01.

^3^ BMI Categories (kg/m^2^): Normal weight, 18.5≤ BMI <25; Overweight, 25≤ BMI <30; Obese, BMI ≥ 30. Underweight (BMI < 18.5, n=144) were excluded due to a small sample size.

^4^ Race/Hispanic Origin does not sum to 100 because the other race category is not shown.
