# Supplementary material for: Examination of different definitions of snacking frequency and associations with weight status among U.S. adults
Source: PLoS One. 2020 Jun 17;15(6):e0234355. doi: 10.1371/journal.pone.0234355 (PMC7299329; doi:10.1371/journal.pone.0234355)
Supplement: S2 Table — 1–2 Abbreviations: Ref, Reference Value. 1 OW/OB: Odds ratio of overweight or obesity (BMI≥25); WC: Odds ratio of waist circumference > 102cm; SAD: Odds ratio of SAD>25cm; EER: Men EER = 662 –(9.53 x age [y]) + PA x [(15.91 x weight [kg]) + (539.6 x height [m])], Women EER = 354 –(6.91 x age [y]) + PA x [(9.36 x weight [kg]) + (726 x height [m])]. A p-value of < 0.001 was considered statistically significant. 2 An asterisk “*”denotes a snack defined as an event that contributed ≥ 50 kcal. 3 Snack defined as an event defined by the reporter as a “snack.” 4 Covariates included in Model 2: age (continuous), race (NH—White, NH-Black, NH-Asian, Hispanic), family income-to-poverty ratio (PIR ≤ 130%, 130% < PIR ≥ 350%, PIR > 350%), day of recall (weekend, weekday), typical intake (usual intake, much more than usual, much less than usual), smoking status (never smoked, former smoker, current smoker occasionally, current smoker daily), and EI:EER. 5 Covariates included in Model 3: covariates included in Model 2 with the exception of EI:EER, and the addition of mean energy of a snack and mean energy density (kcal/g) of a snack. 6 Snack defined as an event outside of a typical mealtime. (DOCX) [file pone.0234355.s003.docx]

**S2 Table. Odds Ratio of Overweight or Obesity (BMI ≥ 25 kg/m^2^), Waist Circumference (WC) >102cm, and Sagittal Abdominal Diameter (SAD) >25cm with Snacking Frequency among U.S. Adult Men (≥20y) in Models Controlling for Additional Covariates, NHANES 2013-2016^1,2^**

|  |  | **BMI** | | **WC** | | **SAD** | |
| --- | --- | --- | --- | --- | --- | --- | --- |
| **Daily Occasions** | **n** | **OR (95% CI)** | **P** | **OR (95% CI)** | **P** | **OR (95% CI)** | **P** |
| **Snacks^3^ - Model 2^4^** | | | | | | | |
| 0 | 1335 | 1.00 (Ref) | - | 1.00 (Ref) | - | 1.00 (Ref) | - |
| 1 | 1534 | 1.11 (0.87, 1.43) | 0.39 | 1.10 (0.84, 1.44) | 0.49 | 1.17 (0.85, 1.59) | 0.32 |
| 2 | 1083 | 1.03 (0.74, 1.41) | 0.87 | 0.83 (0.64, 1.07) | 0.15 | 1.01 (0.72, 1.41) | 0.95 |
| 3 | 490 | 0.97 (0.65, 1.44) | 0.86 | 0.90 (0.66, 1.22) | 0.48 | 1.04 (0.72, 1.51) | 0.81 |
| 4+ | 284 | 1.01 (0.58, 1.75) | 0.97 | 1.19 (0.72, 1.94) | 0.49 | 1.18 (0.74, 1.87) | 0.48 |
| **Snacks^3^ - Model 3^5^** | | | | | | | |
| 0 | 1335 | 1.00 (Ref) | - | 1.00 (Ref) | - | 1.00 (Ref) | - |
| 1 | 1534 | 1.04 (0.80, 1.36) | 0.75 | 0.99 (0.71, 1.37) | 0.94 | 1.13 (0.77, 1.67) | 0.52 |
| 2 | 1083 | 0.89 (0.65, 1.22) | 0.45 | 0.67 (0.48, 0.92) | 0.02 | 0.91 (0.59, 1.41) | 0.67 |
| 3 | 490 | 0.84 (0.58, 1.21) | 0.33 | 0.71 (0.52, 0.99) | 0.04 | 0.96 (0.59, 1.56) | 0.86 |
| 4+ | 284 | 0.76 (0.43, 1.35) | 0.34 | 0.81 (0.47, 1.40) | 0.44 | 0.95 (0.52, 1.75) | 0.88 |
| **Snacks, ≥ 50 kcal occasions^3,*^ - Model 2^4^** | | | | | | | |
| 0 | 1567 | 1.00 (Ref) | - | 1.00 (Ref) | - | 1.00 (Ref) | - |
| 1 | 1619 | 1.12 (0.87, 1.43) | 0.37 | 1.05 (0.84, 1.30) | 0.67 | 1.09 (0.85, 1.40) | 0.49 |
| 2 | 1011 | 1.06 (0.78, 1.43) | 0.72 | 0.76 (0.58, 1.00) | 0.05 | 0.95 (0.68, 1.32) | 0.74 |
| 3 | 361 | 0.99 (0.64, 1.54) | 0.97 | 1.00 (0.75, 1.32) | 0.98 | 1.12 (0.76, 1.63) | 0.56 |
| 4+ | 168 | 1.11 (0.59, 2.09) | 0.74 | 1.11 (0.58, 2.16) | 0.74 | 1.06 (0.60, 1.90) | 0.83 |
| **Snacks, ≥ 50 kcal occasions^3,*^ - Model 3^5^** | | | | | | | |
| 0 | 1567 | 1.00 (Ref) | - | 1.00 (Ref) | - | 1.00 (Ref) | - |
| 1 | 1619 | 0.99 (0.76, 1.29) | 0.94 | 0.87 (0.66, 1.16) | 0.34 | 1.00 (0.72, 1.39) | 1.00 |
| 2 | 1011 | 0.86 (0.60, 1.24) | 0.42 | 0.57 (0.41, 0.79) | 0.002 | 0.80 (0.52, 1.25) | 0.32 |
| 3 | 361 | 0.75 (0.49, 1.15) | 0.19 | 0.68 (0.48, 0.95) | 0.03 | 0.89 (0.56, 1.41) | 0.61 |
| 4+ | 168 | 0.72 (0.36, 1.45) | 0.35 | 0.64 (0.32, 1.27) | 0.19 | 0.72 (0.38, 1.38) | 0.31 |
| **Snacks + other eating between meals^6^ - Model 2^4^** | | | | | | | |
| 0 | 419 | 1.00 (Ref) | - | 1.00 (Ref) | - | 1.00 (Ref) | - |
| 1 | 931 | 1.17 (0.81, 1.70) | 0.39 | 0.96 (0.71, 1.30) | 0.80 | 1.06 (0.63, 1.79) | 0.82 |
| 2 | 1080 | 1.02 (0.65, 1.60) | 0.92 | 0.91 (0.67, 1.24) | 0.54 | 1.06 (0.61, 1.82) | 0.84 |
| 3 | 958 | 1.32 (0.88, 2.00) | 0.17 | 1.18 (0.86, 1.62) | 0.28 | 1.37 (0.80, 2.35) | 0.24 |
| 4+ | 1338 | 1.17 (0.75, 1.81) | 0.48 | 0.95 (0.67, 1.33) | 0.74 | 1.00 (0.56, 1.77) | 0.99 |
| **Snacks + other eating between meals^6^ - Model 3^4^** | | | | | | | |
| 0 | 419 | 1.00 (Ref) | - | 1.00 (Ref) | - | 1.00 (Ref) | - |
| 1 | 931 | 1.14 (0.80, 1.63) | 0.45 | 0.93 (0.67, 1.27) | 0.62 | 1.06 (0.63, 1.78) | 0.82 |
| 2 | 1080 | 1.00 (0.65, 1.54) | 0.99 | 0.84 (0.59, 1.19) | 0.31 | 1.04 (0.60, 1.79) | 0.90 |
| 3 | 958 | 1.18 (0.78, 1.78) | 0.42 | 0.99 (0.67, 1.46) | 0.96 | 1.24 (0.71, 2.15) | 0.44 |
| 4+ | 1338 | 0.96 (0.64, 1.44) | 0.86 | 0.74 (0.50, 1.10) | 0.13 | 0.86 (0.48, 1.55) | 0.61 |
| **Snacks + other eating between meals, ≥50 kcal occasions^6,*^- Model 2^4^** | | | | | | | |
| 0 | 999 | 1.00 (Ref) | - | 1.00 (Ref) | - | 1.00 (Ref) | - |
| 1 | 1386 | 1.30 (1.01, 1.68) | 0.04 | 0.99 (0.77, 1.26) | 0.91 | 1.09 (0.81, 1.46) | 0.57 |
| 2 | 1214 | 1.22 (0.94, 1.59) | 0.12 | 0.93 (0.72, 1.20) | 0.55 | 1.17 (0.85, 1.61) | 0.32 |
| 3 | 683 | 1.23 (0.84, 1.79) | 0.27 | 0.79 (0.55, 1.14) | 0.20 | 1.04 (0.74, 1.47) | 0.81 |
| 4+ | 444 | 1.30 (0.84, 2.01) | 0.23 | 1.11 (0.75, 1.65) | 0.59 | 1.13 (0.72, 1.79) | 0.58 |
| **Snacks + other eating between meals, ≥50 kcal occasions^6,*^ - Model 3^5^** | | | | | | | |
| 0 | 999 | 1.00 (Ref) | - | 1.00 (Ref) | - | 1.00 (Ref) | - |
| 1 | 1386 | 1.19 (0.94, 1.51) | 0.15 | 0.82 (0.63, 1.06) | 0.12 | 0.98 (0.72, 1.34) | 0.91 |
| 2 | 1214 | 1.03 (0.78, 1.36) | 0.83 | 0.68 (0.50, 0.92) | 0.01 | 0.97 (0.68, 1.36) | 0.84 |
| 3 | 683 | 0.99 (0.65, 1.51) | 0.96 | 0.55 (0.35, 0.84) | 0.01 | 0.83 (0.53, 1.29) | 0.39 |
| 4+ | 444 | 0.87 (0.56, 1.36) | 0.54 | 0.62 (0.39, 0.98) | 0.04 | 0.76 (0.45, 1.27) | 0.28 |

Abbreviations: Ref, Reference Value.

^1^ OW/OB: Odds ratio of overweight or obesity (BMI≥25); WC: Odds ratio of waist circumference > 102cm; SAD: Odds ratio of SAD>25cm; EER: Men EER = 662 – (9.53 x age [y]) + PA x [(15.91 x weight [kg]) + (539.6 x height [m])], Women EER = 354 – (6.91 x age [y]) + PA x [(9.36 x weight [kg]) + (726 x height [m])]. A *p*-value of < 0.001 was considered statistically significant.

^2^ An asterisk “*”denotes a snack defined as an event that contributed ≥ 50 kcal.

^3^ Snack defined as an event defined by the reporter as a “snack.”

^4^ Covariates included in Model 2: age (continuous), race (NH - White, NH-Black, NH-Asian, Hispanic), family income-to-poverty ratio (PIR ≤ 130%,

130% < PIR ≥ 350%, PIR > 350%), day of recall (weekend, weekday), typical intake (usual intake, much more than usual, much less than usual), smoking status (never smoked, former smoker, current smoker occasionally, current smoker daily), and EI:EER.

^5^ Covariates included in Model 3: covariates included in Model 2 with the exception of EI:EER, and the addition of mean energy of a snack and mean energy density (kcal/g) of a snack.

^6^ Snack defined as an event outside of a typical mealtime.
